# Efficacy and safety of sarolaner (Simparica^®^) in the treatment and control of naturally occurring flea infestations in dogs presented as veterinary patients in Australia

**DOI:** 10.1186/s13071-017-2321-3

**Published:** 2017-08-16

**Authors:** Raj Packianathan, Sally Colgan, Andrew Hodge, Kylie Davis, Robert H. Six, Steven Maeder

**Affiliations:** 1 Zoetis, Veterinary Medicine Research and Development, Level 6, 5 Rider Boulevard, Rhodes, NSW 2138 Australia; 2 Eurofins SCEC, PO Box 211, Northbridge, NSW 1560 Australia; 3 Zoetis, Veterinary Medicine Research and Development, 333 Portage St, Kalamazoo, MI 49007 USA

**Keywords:** Flea, Sarolaner, Simparica, *Ctenocephalides felis*, Efficacy, Dogs, Isoxazoline, Field study, Spinosad, Parasiticide, Oral

## Abstract

**Background:**

The efficacy and safety of a novel isoxazoline compound, sarolaner (Simparica^®^, Zoetis) and spinosad (Comfortis^®^, Elanco) as a positive control were evaluated for the treatment and control of natural flea infestations on dogs in two randomised, blinded, multi-centric clinical trials conducted in 11 veterinary clinics in northeastern and southeastern states of Australia.

**Methods:**

A total of 162 client-owned dogs (80 in northern study and 82 in southern study) from 105 households were enrolled. Each household was randomly allocated to receive either sarolaner (Simparica^®^, Zoetis) or spinosad (Comfortis^®^, Elanco). Dogs were dosed on Days 0, 30 and 60 and physical examinations and flea counts were conducted on Days 0, 14, 30, 60 and 90. Efficacy assessments were based on the percentage reduction in live flea counts post-treatment compared to Day 0.

**Results:**

In the northern study, at enrolment, primary dogs had flea counts ranging from 5 to 772. At the first efficacy assessment on Day 14, sarolaner resulted in 99.3% mean reduction in live flea counts relative to Day 0, compared to 94.6% in the spinosad group. On Day 30, the sarolaner-treated group had mean efficacy of 99.2% compared to 95.7% in the spinosad-treated group, and on days 60 and 90, both groups had mean efficacies of ≥ 98.8%. In the southern study, at enrolment, primary dogs had flea counts ranging from 5 to 156. Both sarolaner and spinosad resulted in ≥ 96.7% mean reduction in live flea counts on Day 14. On Day 30, the sarolaner-treated group had mean efficacy of 99.5% compared to 89.7% in the spinosad-treated group, and on days 60 and 90, both groups had mean efficacies of ≥ 98.6%. No treatment-related adverse events were observed in either study.

**Conclusions:**

A single monthly dose of sarolaner (Simparica^®^) administered orally at 2–4 mg/kg for three consecutive months was well tolerated and provided excellent efficacy against natural infestations of fleas under a range of Australian field conditions including different climatic and housing conditions. Similar efficacy was observed with spinosad (Comfortis^®^) after the second and third monthly treatments.

## Background

The cat flea, *Ctenocephalides felis felis*, is the most common ectoparasite of dogs and cats worldwide [1]. In Australia, *C. felis felis* is the most common flea species in domestic dogs and *Ctenocephalides canis* and *Echidnophaga gallinacea* have also been reported in western Australia [2, 3]. The *C. felis* in Australia is believed to have limited genetic diversity [2]; however, two different haplotype clades among *C. felis* in the north and south eastern part of Australia have been reported using mitochondrial DNA markers [2, 4, 5]. *Ctenocephalides felis* infests domestic dogs and cats causing irritation, discomfort and flea allergy dermatitis due to flea bites. Flea allergy dermatitis is one of the most common skin conditions of dogs in warm humid parts of Australia [6]. Fleas also act as an intermediate host for the dog tapeworm, *Dipylidium caninum* and they can transmit a number of pathogens in domestic animals and humans, including *Rickettsia felis*, *Bartonella clarridgeiae* [3, 7, 8] and *Bartonella henselae* [9]. Year-round prophylaxis is the gold standard for control of fleas on dogs and cats. Over the years, treatment and control of fleas on companion animals have been revolutionized by the introduction of new classes of ectoparasiticides targeting adult and intermediate stages of fleas [6]. Insecticide resistance to cat fleas is not thoroughly understood [10]. Despite the general belief that perceived lack of efficacy seen in the field against some of well-known actives is due to incorrect treatment administration or client non-compliance, resistance to some of the older and well-known actives is thought to exist [11].

Sarolaner is a novel isoxazoline compound developed by Zoetis as an oral ectoparasiticide with a broad spectrum of activity against fleas, ticks and mites in dogs [12, 13]. Sarolaner has demonstrated efficacy against both *C. felis* including the KS1 strain and *C. canis* [14, 15], many different species of ticks [16–18] including the Australian paralysis tick, *Ixodes holocyclus* [19] and mites including *Sarcoptes scabiei*, *Demodex* spp. and *Otodectes cynotis* [20, 21].

A single monthly dose of sarolaner administered at a minimum of 2 mg/kg provides excellent efficacy against fleas for a period of 35 days with a speed of kill as early as 3 h after treatment [22]. Here we report on two multi-centric field studies to evaluate the efficacy and safety of sarolaner (Simparica^®^, Zoetis) oral tablets administered at 2–4 mg/kg compared to spinosad oral tablets (Comfortis^®^, Elanco) as a positive control administered per label, for the treatment and prevention of natural flea infestations in client-owned dogs in Australia.

## Methods

The studies were conducted in accordance with the World Association for the Advancement of Veterinary Parasitology (WAAVP) guidelines for evaluating the efficacy of parasiticides for the treatment, prevention and control of flea and tick infestation on dogs and cats [23] and complied with Good Clinical Practices [24]. The protocols were reviewed and approved by the Animal Care and Ethics Committee of the Director-General of NSW Department of Primary Industries.

### Study locations

**Fig. 1:**
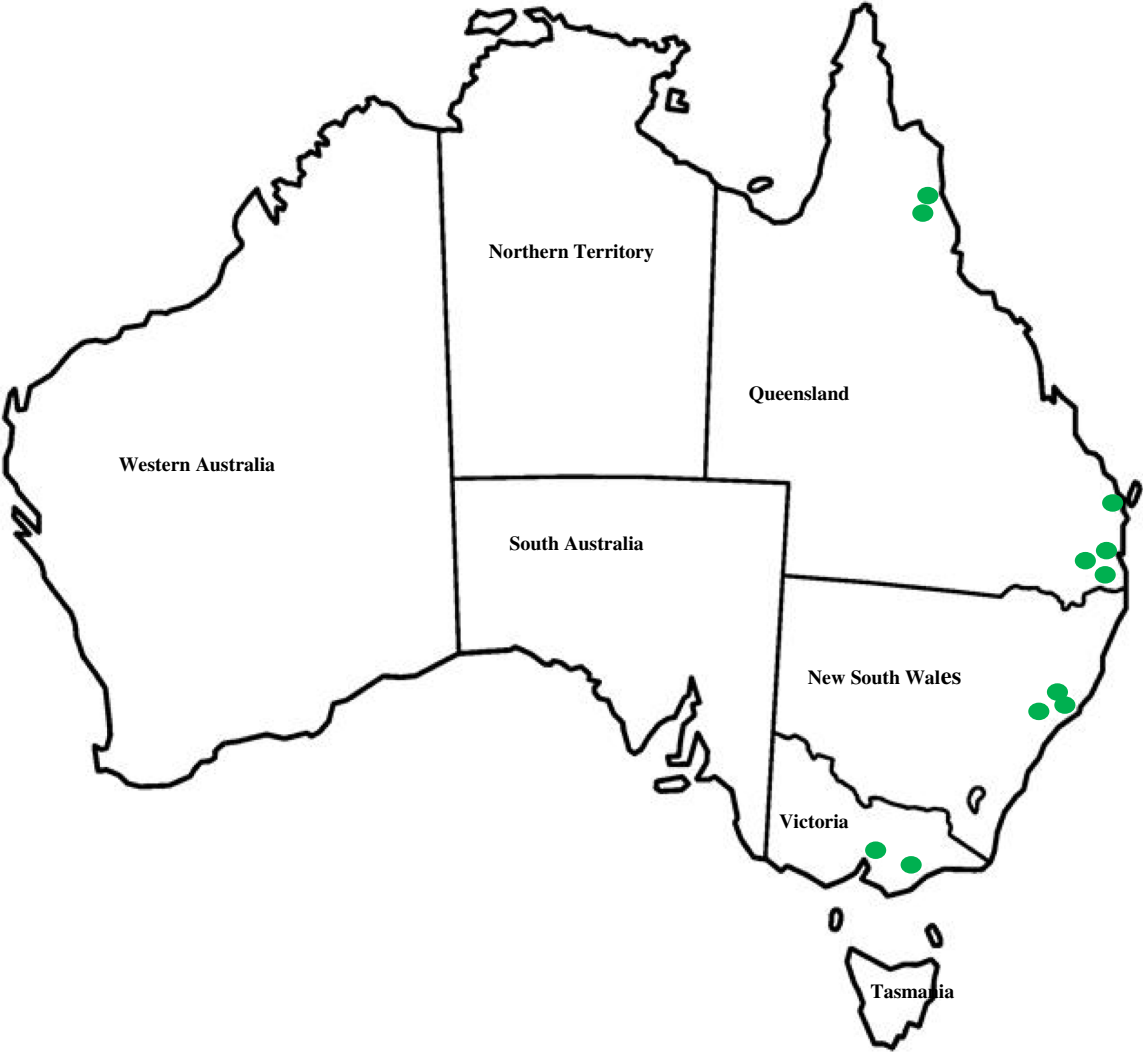
Clinic locations of dogs enrolled in two clinical field studies in the northern and southern regions of Australia

Two field studies were conducted, one in northeastern (northern study) and one in southeastern (southern study) Australia. Northeastern Australia (Queensland) has a subtropical climate compared to the temperate climate in southeastern Australia (New South Wales and Victoria). The number and location of enrolled veterinary clinics in the different regions are summarised in Fig. 1 and Table 1.

**Table 1:** Clinic location and number of dogs enrolled in two clinical field studies in the northern and southern regions of Australia

Clinic location	Primary dogs (efficacy)			All dogs (safety)		
	Sarolaner (2–4 mg/kg)	Spinosad (≥ 30 mg/kg)	Total	Sarolaner (2–4 mg/kg)	Spinosad (≥ 30 mg/kg)	Total
Northern study						
Atherton, QLD	5	2	7	12	3	15
Indooroopilly, QLD	4	2	6	6	2	8
Stratford, QLD	9	5	14	12	7	19
Hervey Bay, QLD	12	7	19	18	8	26
New Farm, QLD	2	1	3	4	1	5
Kuraby, QLD	3	2	5	3	4	7
Total	35	19	54	55	25	80
Southern study						
St Marys, NSW	2	1	3	2	1	3
Northbridge, NSW	6	4	10	14	7	21
Guildford, NSW	12	6	18	17	11	28
Trafalgar, VIC	6	3	9	8	6	14
Moorabbin, VIC	7	4	11	9	7	16
Total	33	18	51	50	32	82

### Animals

Client-owned dogs recruited from veterinary clinics were used in the studies. Dogs were from diverse households and lived both indoors and outdoors. Dogs came from both single dog households and households with multiple dogs (maximum of 3 dogs) and/or cats. There were no breed or sex restrictions, but dogs intended for breeding or that were pregnant or lactating were not eligible for enrolment. For inclusion in the study, at least one dog in the household had to harbor at least 5 live fleas at screening. All dogs were at least 8 weeks of age and ≥ 1.3 kg in body weight at enrolment. Dogs aged less than 14 weeks or with a body weight of ≤ 2.3 kg and allocated to the spinosad treatment group were excluded from the study to comply with Comfortis^®^ label recommendations. Dogs in the study were not allowed to have been treated with any ectoparasiticide with persistent activity within 30 days or with a short-acting ectoparasiticide within 14 days of the first treatment.

### Experimental design

The northern and southern studies were analysed and reported separately. Within each region, the study was a multi-centric, blinded, positively-controlled trial with a randomized block design. Households were allocated randomly to treatment with sarolaner or spinosad in a ratio of 2:1 based on the order of enrollment. Within each household, one dog was randomly nominated as the primary dog and up to 2 additional dogs were enrolled as supplementary dogs. All enrolled dogs from the same household received the same treatment as the primary dog. Only the primary dogs were included in the efficacy evaluation whereas all dogs were included in the safety evaluation.

All dogs were confirmed to be in good general health prior to enrollment based on the physical examination performed by a veterinarian. Dogs were housed and maintained under their normal home conditions for the duration of the study.

Because sarolaner has efficacy against Australian tick species (*I. holocyclus* and *Rhipicephalus sanguineus*) for up to 35 days, sarolaner-treated dogs were not allowed to receive any other product for tick control. For the spinosad-treated dogs, amitraz collar (Preventic^®^, Virbac Australia) was allowed as an optional tick control if indicated. The clients were requested to remove any tick collars on all dogs prior to each veterinary clinic visit in order to maintain the blinding of treatment groups. All cats in the households were treated with commercially available flea products.

No additional products (systemic, premise, and/or over-the-counter treatments including insecticidal shampoos or collars) that had activity against fleas were permitted to be used on any animal in the household for the duration of the study. Any concomitant medications used during the study were recorded along with any abnormal health events.

### Treatment administration

Day 0 was set as the day the primary dog in each household received the first treatment. Dogs received three consecutive monthly treatments on study days 0, 30 and 60. For the follow-up treatments on Days 30 and 60, the visits were allowed to deviate by ± 3 days of the target date. All treatments were dispensed according to a randomization plan that was provided for each clinic before study start. Treatment dispensing was based upon the most recent body weight. Treatments were administered by an unmasked study participant (the dispenser) at the clinics in presence of the owners. Animals enrolled in the sarolaner group were treated with the appropriate strength sarolaner chewable tablet (Simparica^®^, Zoetis) to provide the recommended minimum dosage of 2 mg/kg (range 2–4 mg/kg). There were no restrictions regarding the prandial state at the time of sarolaner administration, therefore tablets could be administered with or without food. Dogs enrolled in the positive control group received spinosad (Comfortis^®^ Chewable Tablets, Elanco), according to the manufacturer’s label recommendations to deliver ≥ 30 mg/kg spinosad. Spinosad was administered with a small meal in order to comply with the approved dosing directions for that product.

### Flea counts

Flea counts on primary dogs were conducted prior to treatment on Day 0, and on post-treatment Days 14, 30, 60 and 90 (the post-treatment evaluations could be conducted ± 3 days of the target day) by a veterinarian.

Flea counts were conducted by personnel trained to a standardized methodology. The dog was combed with a fine toothed flea comb that was uniquely identified for each dog. The combing proceeded in a systematic manner to ensure all areas of the dog were combed. Each dog was examined for at least 10 minutes. If any fleas were found in the last 5 minutes, the examination was continued in 5 minute increments until no fleas were encountered. All fleas were removed from the dog and discarded after counting. Fleas maintaining an upright orientationor moving in a coordinated manner were considered to be live. Only live flea counts were recorded.

### Statistical analysis

Data were summarised and analysed for each of the two studies separately, using SAS version 9.3 (SAS Institute Inc., Cary, NC, USA). The individual animal (primary dog) was the experimental unit for the efficacy analysis and all treatment comparisons were carried out at the 5% significance level (two-sided).

Data were excluded from the efficacy analysis following protocol deviations such as incorrect dosing or where dosing or flea counts were not conducted within ± 3 days of the target day (after Day 0).

Percent efficacy (percentage reduction in live flea counts from pre-treatment count on Day 0) was calculated for each animal at each time point after Day 0. Percent efficacy was analyzed using a general linear mixed model for repeated measures with fixed effects for treatment, time and the treatment by time interaction. The random effects included clinic, the interaction between clinic and treatment, block within clinic, animal within block, treatment and clinic, the interaction of clinic, treatment and time, and residual. Least squares means were used as estimates of the treatment means at each time point.

## Results

### Demographics

In both studies, the treatment groups were generally well-balanced in terms of the various demographic characteristics. Enrolment and demographic characteristics are summarised in Tables 1, 2 and 3.

**Table 2:** Demographic characteristics of dogs enrolled in two clinical field studies in the northern and southern regions of Australia

Characteristic	Northern study		Southern study	
	Sarolaner (2–4 mg/kg)	Spinosad (≥ 30 mg/kg)	Sarolaner (2–4 mg/kg)	Spinosad (≥ 30 mg/kg)
	(*n* = 55)	(*n* = 25)	(*n* = 50)	(*n* = 32)
Breed				
Purebred	19 (35%)	11 (44%)	26 (52%)	12 (38%)
Non-purebred	36 (65%)	14 (56%)	24 (48%)	20 (63%)
Living condition				
Indoors and outdoors	30 (55%)	16 (64%)	18 (36%)	13 (41%)
Mostly indoors	10 (18%)	3 (12%)	9 (18%)	6 (19%)
Mostly outdoors	15 (27%)	6 (24%)	23 (46%)	13 (41%)
Sex				
Male	23 (42%)	14 (56%)	24 (48%)	20 (63%)
Female	32 (58%)	11 (44%)	26 (52%)	12 (38%)
Neutered				
Yes	42 (76%)	19 (76%)	30 (60%)	22 (69%)
No	13 (24%)	6 (24%)	20 (40%)	10 (31%)
Hair type				
Long	5 (9%)	5 (20%)	6 (12%)	3 (9%)
Medium	19 (35%)	5 (20%)	19 (38%)	16 (50%)
Short	31 (56%)	15 (60%)	25 (50%)	13 (41%)

**Table 3:** Age and weight of dogs at enrolment in two clinical field studies in the northern and southern regions of Australia

Characteristic	Northern study		Southern study	
	Sarolaner (2–4 mg/kg)	Spinosad (≥ 30 mg/kg)	Sarolaner (2–4 mg/kg)	Spinosad (≥ 30 mg/kg)
	(*n* = 55)	(*n* = 25)	(*n* = 50)	(*n* = 32)
Age (years)				
Mean	4.7	4.8	5.5	5.9
Range	0.3–14.0	0.5–14.0	0.3–15.0	0.5–14.0
Body weight (kg)				
Mean	18.3	21.6	18.6	14.4
Range	2.6–50.8	5.8–40.4	1.8–56.0	2.7–38.0

#### Northern study

A total of 80 dogs (54 primary dogs) were enrolled across 6 different clinics in Queensland. Of the total of 80 dogs, 30 (38%) were purebred and 50 (62%) were cross-bred. The majority of the dogs were neutered (76%) with approximately equal numbers of males and females. Out of 54 households, 41 households had dogs only and 13 had both dogs and cats. The age of the enrolled dogs ranged from 16 weeks to 14 years, with a mean age of 4.7 years. Eighty eight percent (88%) of enrolled dogs had short or medium hair length and the majority (84%) had outdoor access, with only 16% living mainly indoors. Two dogs in each treatment group were withdrawn from the study. The dogs in the sarolaner-treated group were withdrawn due to owner non-compliance and re-homing of the primary study dog. Two dogs in the spinosad-treated group were withdrawn from the study because of treatment with an anti-parasitic drug prohibited in the study protocol.

#### Southern study

A total of 82 dogs (51 primary dogs) were enrolled across 5 different clinics in NSW and Victoria. Of the total of 82 dogs, 38 (46%) were purebred and 44 (54%) were cross-bred. The majority of the dogs were neutered (63%). The age of the enrolled dogs ranged from 16 weeks to 15 years, with a mean age of 5.7 years. Eighty nine percent (89%) of enrolled dogs had short or medium hair length and the majority (82%) had outdoor access, with only 18% living mainly indoors. Out of 51 households, 25 households had dogs only and 26 had both dogs and cats. A total of 7 dogs in the sarolaner-treated group were withdrawn from the southern study due to medical conditions (3 dogs) or owner non-compliance (4 dogs).

### Flea counts

#### Northern study

At enrolment, primary dogs had flea counts ranging from 5 to 772 (Table 4). Sarolaner resulted in 99.3% mean reduction in live flea counts compared to 94.6% in the spinosad group on Day 14. At subsequent efficacy measurements on Days 30, 60 and 90, both treatments resulted in ≥ 95.7% mean reduction in live flea counts.

**Table 4:** Flea counts, ranges of counts and mean efficacy at each time point for primary dogs in two clinical field studies in the northern and southern regions of Australia

Day of study	Northern study		Southern study	
	Sarolaner (2–4 mg/kg)	Spinosad (≥ 30 mg/kg)	Sarolaner (2–4 mg/kg)	Spinosad (≥ 30 mg/kg)
Day 0				
No. of animals	34	19	33	18
Arithmetic mean count	39.5	127.9	27.6	47.8
Range of counts	7–288	5–772	5–119	5–156
Day 14				
No. of animals	34	19	31	16
Arithmetic mean count	0.4	3.9	1.1	2.3
Range of counts	0–7	0–32	0–18	0–28
Mean efficacy (%)^a^	99.3	94.6	96.7	97.6
Range of efficacy (%)^a^	83–100	72–100	50–100	82–100
Day 30				
No. of animals	32	18	29	18
Arithmetic mean count	0	7.9	0.2	10.7
Range of counts	0–1	0–103	0–3	0–130
Mean efficacy (%)^a^	99.2	95.7	99.5	89.7
Range of efficacy (%)^a^	90–100	73–100	96–100	0–100
Day 60				
No. of animals	30	18	27	16
Arithmetic mean count	0	0	0.1	0
Range of counts	0–1	0–0	0–2	0–0
Mean efficacy (%)^a^	98.8	100	98.6	100
Range of efficacy (%)^a^	92–100	100–100	60–100	100–100
Day 90				
No. of animals	29	15	23	16
Arithmetic mean count	0	0	0	0.4
Range of counts	0–0	0–0	0–0	0–4
Mean efficacy (%)^a^	99.9	100	100	99.3
Range of efficacy (%)^a^	100–100	100–100	100–100	92–100

^a^Efficacy calculated for each animal as the percentage reduction in flea count compared to Day 0

*Note*: In both studies, there was no significant evidence of an overall difference between treatment groups, with non-significant terms for the treatment group main effect and the treatment by time interaction term in the repeated measures models (*P* > 0.14 in all cases). Therefore, comparisons between the treatment groups at each time point have not been presented

#### Southern study

At enrolment, primary dogs had flea counts ranging from 5 to 156 (Table 4). Both sarolaner and spinosad resulted in ≥ 96.7% mean reduction in live flea counts on Day 14. On Day 30, the sarolaner-treated group had mean efficacy of 99.5% compared to 89.7% in the spinosad-treated group. On day 60, the sarolaner-treated group had mean efficacy of 98.6% compared to 100% in the spinosad-treated group. On Day 90, the sarolaner-treated group had mean efficacy of 100% compared to 99.3% in the spinosad-treated group.

There was no significant difference between the mean efficacies of the two treatment groups in either study on any post-treatment day (*P* > 0.14).

### Safety

There were no treatment-related adverse events in sarolaner or spinosad-treated dogs. The majority of the observed adverse events in both studies were sporadic in nature and typical of those commonly seen in the general dog population such as skin, gastrointestinal, eye, ear, musculoskeletal and systemic conditions. These adverse events were observed in 14 sarolaner-treated dogs (25%) and 10 spinosad-treated dogs (40%) in the northern study and 21 sarolaner-treated dogs (42%) and 20 spinosad-treated dogs (63%) in the southern study. The overall incidence of these adverse events are summarised in Table 5.

**Table 5:** Incidence of adverse events occurring in ≥ 2% of sarolaner-treated dogs presented as veterinary patients following once a month dosing with sarolaner or spinosad for three months

Adverse events	Northern study		Southern study	
	Sarolaner (2–4 mg/kg)	Spinosad (≥ 30 mg/kg)	Sarolaner (2–4 mg/kg)	Spinosad (≥ 30 mg/kg)
	(*n* = 55)	(*n* = 25)	(*n* = 50)	(*n* = 32)
Skin conditions	6 (11%)	5 (20%)	11 (22%)	13 (41%)
Gastrointestinal conditions	5 (9%)	4 (16%)	4 (8%)	3 (9%)
Eye conditions	1 (2%)	0 (0%)	5 (10%)	4 (13%)
Ear conditions	0 (0%)	0 (0%)	6 (12%)	3 (9%)
Systemic conditions	6 (11%)	2 (8%)	2 (4%)	1 (3%)
Musculo-skeletal conditions	2 (4%)	1 (4%)	0 (0%)	2 (6%)
Any adverse event	14 (25%)	10 (40%)	21 (42%)	20 (63%)

Severe adverse events occurred in one dog in the spinosad group (northern study) and 3 dogs in the sarolaner group (southern study). The spinosad-treated dog developed tick paralysis on Day 36 and was successfully treated and subsequently withdrawn from the study. Of the 3 dogs in the sarolaner group, one primary dog died due to a pre-existing heart condition. At enrolment, this dog had a grade 5/5 heart murmur and abnormal breathing pattern. Approximately 12 h prior to the death on Day 58, the dog was reported to have difficulty breathing. Although a necropsy was not performed to determine the definitive cause of death, the clinical signs strongly support pre-existing cardiopulmonary pathology. The second dog was withdrawn from the study on Day 58 due to congenital pulmonary arterial hypoplasia and secondary ischemia confirmed by cardiac ultrasound examination and magnetic resonance imaging. The third dog was euthanized due to severe arthritis. All of the adverse events were deemed unlikely to be related to treatment administration.

## Discussion

One hundred and sixty-two dogs from 105 different households were enrolled across the two studies. Demographic characteristics of dogs in both studies were similar. Sarolaner (Simparica^®^, Zoetis) chewable tablets administered orally once a month for 3 consecutive months at a minimum dose of 2 mg/kg (dose range of 2–4 mg/kg) resulted in excellent treatment and control of naturally occurring flea infestations on client-owned dogs. Sarolaner and spinosad were well tolerated, with the observed abnormal clinical signs consistent with conditions commonly seen in the general dog population, and not related to study treatment.

Under field conditions, the dogs are continuously exposed to the risk of flea infestation and therefore the rapid onset of immediate and sustained speed of kill is critical in reducing the flea burden on the dogs as well as breaking flea life-cycle and halting their reproduction in the environment [6, 25].

Sarolaner provided 99.3% (northern study) and 96.7% (southern study) mean efficacy at 14 days after the first treatment and these findings were consistent with previous data reported for field studies conducted in the United States and Europe [26, 27]. At the same time point, spinosad provided 94.6 and 97.6% mean efficacy in the northern and southern studies, respectively, which is similar to that previously reported in other studies [26–28]. The efficacy of sarolaner at the end of the first monthly treatment was **≥ **99.2% compared to the spinosad efficacy of ≥ 89.7%. The observed differences in the efficacy between sarolaner- and spinosad-treated dogs following the first monthly treatment were consistent with previous reports [27–29].

Both sarolaner and spinosad demonstrated excellent efficacy after two monthly treatments (≥ 98.6 and ≥ 99.3%, respectively). The mean number of fleas on sarolaner-treated dogs after each monthly treatment ranged from 1 to 3, compared to 0–130 in spinosad-treated dogs. These results are consistent with a previous study [27]. Although identification of flea species in the field has not been conducted in these studies, it is well known that *C. felis felis* is the most common cause of flea infestations in dogs in Australia [2]. Using the mtDNA sequencing of cytochrome *c* oxidase subunits, different haplotype clades of *C. felis* were identified in northeastern and southeastern Australia [4]. Although the acaricidal efficacy against different haplotype clades of *C. felis* is unknown, a proportion of enrolled dogs in both studies (Table 1 and Fig. 1) came from the regions where the different haplotype clades of *C. felis* has been reported [4]. However, there was no apparent difference in the efficacy of either product in areas suspected to have these genetically different populations of *C. felis*.

## Conclusion

Sarolaner (Simparica^®^) administered orally at 2–4 mg/kg once monthly for three consecutive months provided excellent efficacy against natural infestations of fleas on dogs under a range of Australian field conditions. Similar efficacy was observed with spinosad (Comfortis^®^), after the second and third monthly treatments.

## Abbreviations

NSW: New South Wales
QLD: Queensland
VIC: Victoria
